# Supplementary material for: 5hmC enhances PARP trapping and restores PARP inhibitor sensitivity in chemoresistant BRCA1/2-deficient cells
Source: J Biol Chem. 2025 Jun 19;301(7):110393. doi: 10.1016/j.jbc.2025.110393 (PMC12281170; doi:10.1016/j.jbc.2025.110393)

Supplementary Information

Supplementary Figure 1. 5hmC and VitC enhances PARP inhibitor sensitivity in BRCA1/2 deficient cells.

A, B, C. Clonogenic survival assay (A), quantitation of clonogenic survival assay (B) and XTT survival assay (C) was performed in MEF-WT/BRCA1KO cells treated with increasing concentration of olaparib combined with or without VitC (1 mM). N = 3. **D.** Quantitative RT-PCR showing *BRCA1* and *BRCA2* mRNA levels in DLD1 cells after their transient knockdown. **E, F.** Clonogenic survival assay (E) and quantitation of clonogenic survival assay (F) was performed in DLD1 cells treated with indicated drugs upon transient knockdown of either *BRCA1* or *BRCA2*. N = 3. **G, H, I.** Clonogenic survival assay (G), quantitation of clonogenic survival assay (H) and XTT survival assay (I) was performed in UWB1.289-WT/BRCA1KO cells treated with different cytosine analogues (Cytidine, 5mC, 5hmC, 5fC and 5caC) combined with olaparib (100 nM). N = 3. **J, K, L.** Clonogenic survival assay (J), quantitation of clonogenic assay (K) and XTT survival assay (L) was performed in DLD1-WT/BRCA2KO cells treated with cytosine analogues combined with olaparib (100 nM). Data in XTT assay is mean ± SD from 8 technical replicates. Values in XTT assay have been normalized with respective DMSO control of cell line. Statistical analysis was performed using paired T test. ns $P \geq 0.05$, *$P \leq 0.05$, **$P \leq 0.01$, ***$P \leq 0.001$, and ****$P \leq 0.0001$.

Supplementary Figure 1

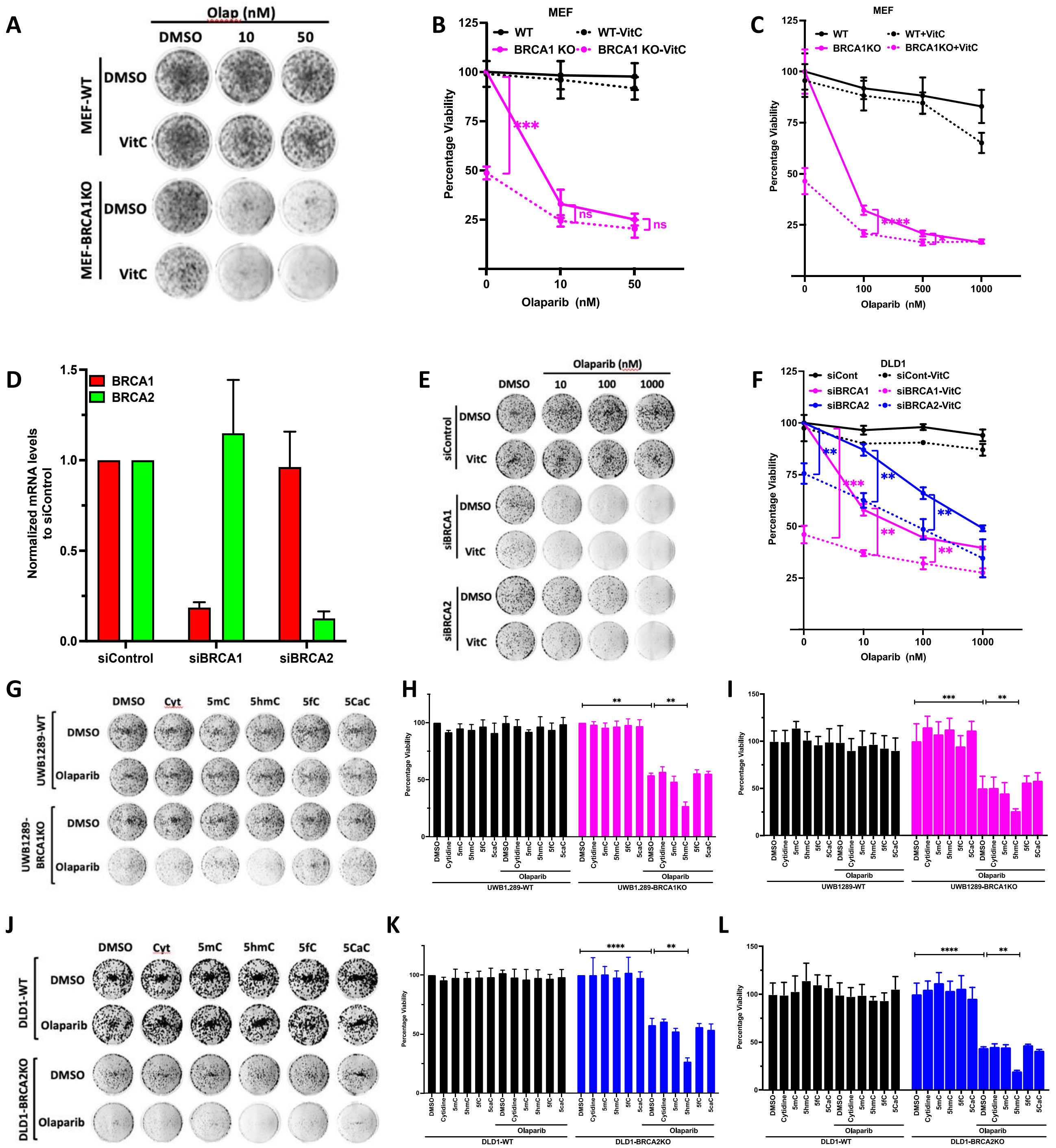

Supplementary Figure 2. 5hmC and VitC restores PARP inhibitor sensitivity in chemoresistant BRCA1/2 deficient cells.

A. Quantitative RT-PCR showing *BRCA1* and *53BP1* mRNA levels in DLD1 cells after their transient knockdown. Values were normalized with respective siControl. **B, C, D.** Clonogenic survival assay (B), quantitation of clonogenic survival assay (C) and XTT survival assay (D) was performed in DLD1 cells treated with increasing concentration of olaparib combined with VitC upon transient knockdown of either *BRCA1* alone or combined with *53BP1*. N = 3. **E.** Quantitative RT-PCR showing *CHD4* and *PTIP* mRNA levels in DLD1-BRCA2KO cells after transient knockdown of *CHD4* or *PTIP*. Values were normalized with respective siControl. **F.** XTT survival assay was performed in WT/*Brca1Δ11*/*Brca1Δ11;53Bp1KO* MEF cells treated with different cytosine analogues (Cytidine, 5mC, 5hmC, 5fC and 5caC) combined with olaparib (100 nM). N = 3. **G, H, I.** Clonogenic survival assay (G), quantitation of clonogenic survival assay (H) and XTT survival assay (I) was performed in DLD1 cells treated with increasing concentration of olaparib combined with 5hmC upon transient knockdown of either *BRCA1* alone or combined with *53BP1*. N = 3. Data in all XTT assays is mean ± SD from 8 technical replicates. Values in XTT assays have been normalized with respective DMSO control of cell line65. Statistical analysis was performed using paired T test. ns $P \geq 0.05$, *$P \leq 0.05$, **$P \leq 0.01$, ***$P \leq 0.001$, and ****$P \leq 0.0001$.

Supplementary Figure 2

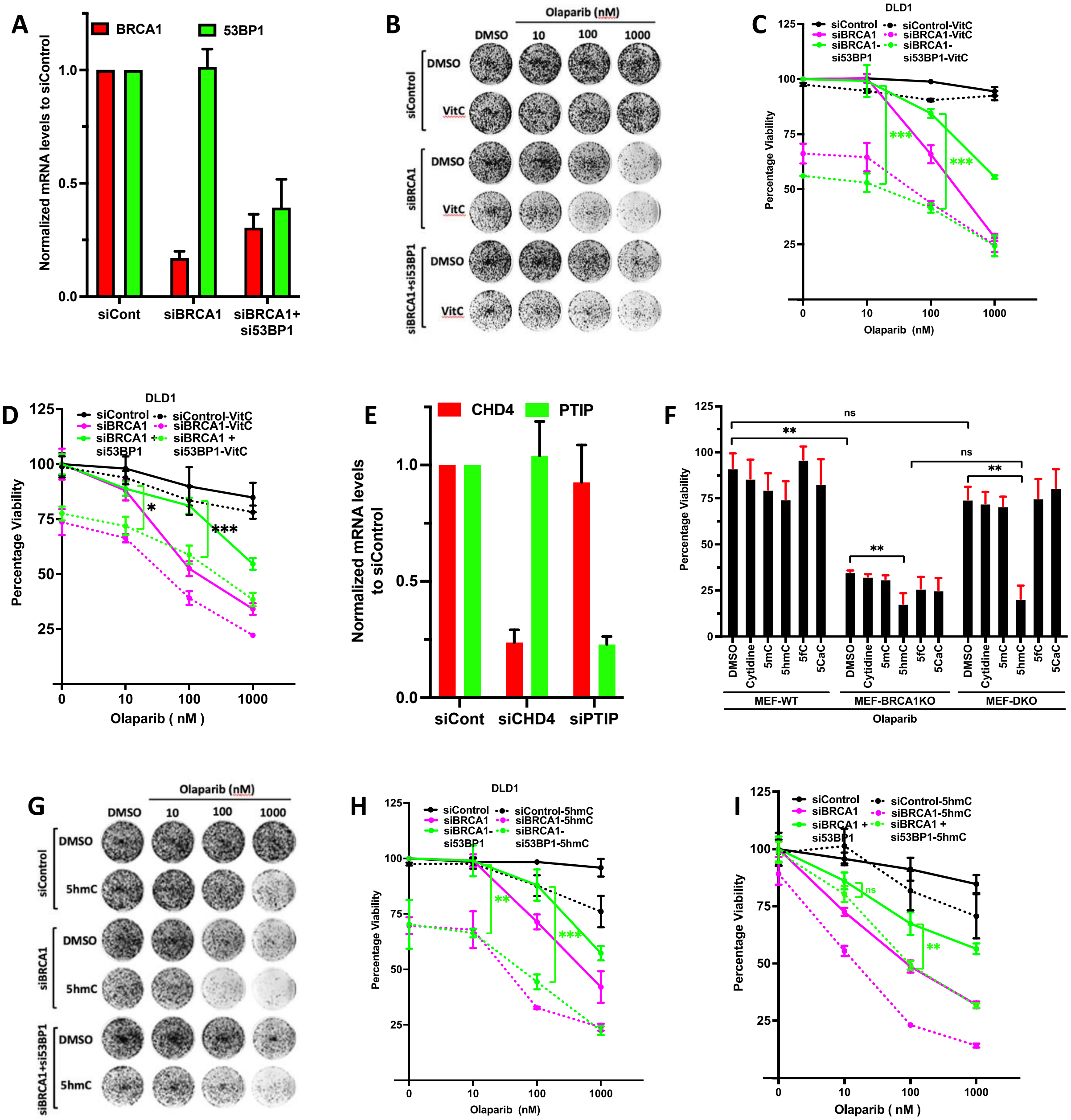

Supplementary Figure 3. VitC enhances the PARP inhibitors sensitivity in BRCA1/2-deficient cells in a TET dependent manner.

A, B. Measurement of ROS levels in UWB 1.289 WT/BRCA1KO (A) and DLD1 WT/BRCA2KO (B) cells upon treatment with VitC (1 mM) or Olaparib (100 nM) or both for 48 hours. Data are means ± SD of 3 technical replicates. Values have been normalized to respective DMSO controls of WT cells. N = 2. **C, D, E.** Mass spectrometry–based quantitation of 5mC levels, as a ratio to that of cytosine in UWB1.289-WT/BRCA1KO (C), DLD1-WT/BRCA2KO (D) and WT/*Brca1Δ11*/*Brca1Δ11;53Bp1KO* MEF (E) cells upon treatments with VitC (1 mM, 48 hrs), olaparib (100 nM, 48 hrs) or both. **F, G, H**. Mass spectrometry–based quantitation of 5hmC levels, as a ratio to that of cytosine, in UWB1.289-WT/BRCA1KO (F) and DLD1-WT/BRCA2KO (G) and WT/*Brca1Δ11*/*Brca1Δ11;53Bp1KO* (DKO) MEF (H) cells upon treatments with olaparib (100 nM, 48 hrs). Mass spectrometry–based data are means ± SD of 3 technical replicates. Values have been normalized to DMSO control of WT cells. N = 2. . **I, J.** Quantitative RT-PCR showing BRCA1/2 and TET1/2/3 mRNA levels in DLD1 cells after transient knockdown of *BRCA1* (I) and *BRCA2* (J) combined with *TET1, 2* and *3*. Values were normalized with respective siControl. Statistical analysis was performed using paired T test. N = 2. ns $P \geq 0.05$, *$P \leq 0.05$, **$P \leq 0.01$, ***$P \leq 0.001$, and ****$P \leq 0.0001$.

Supplementary Figure 3

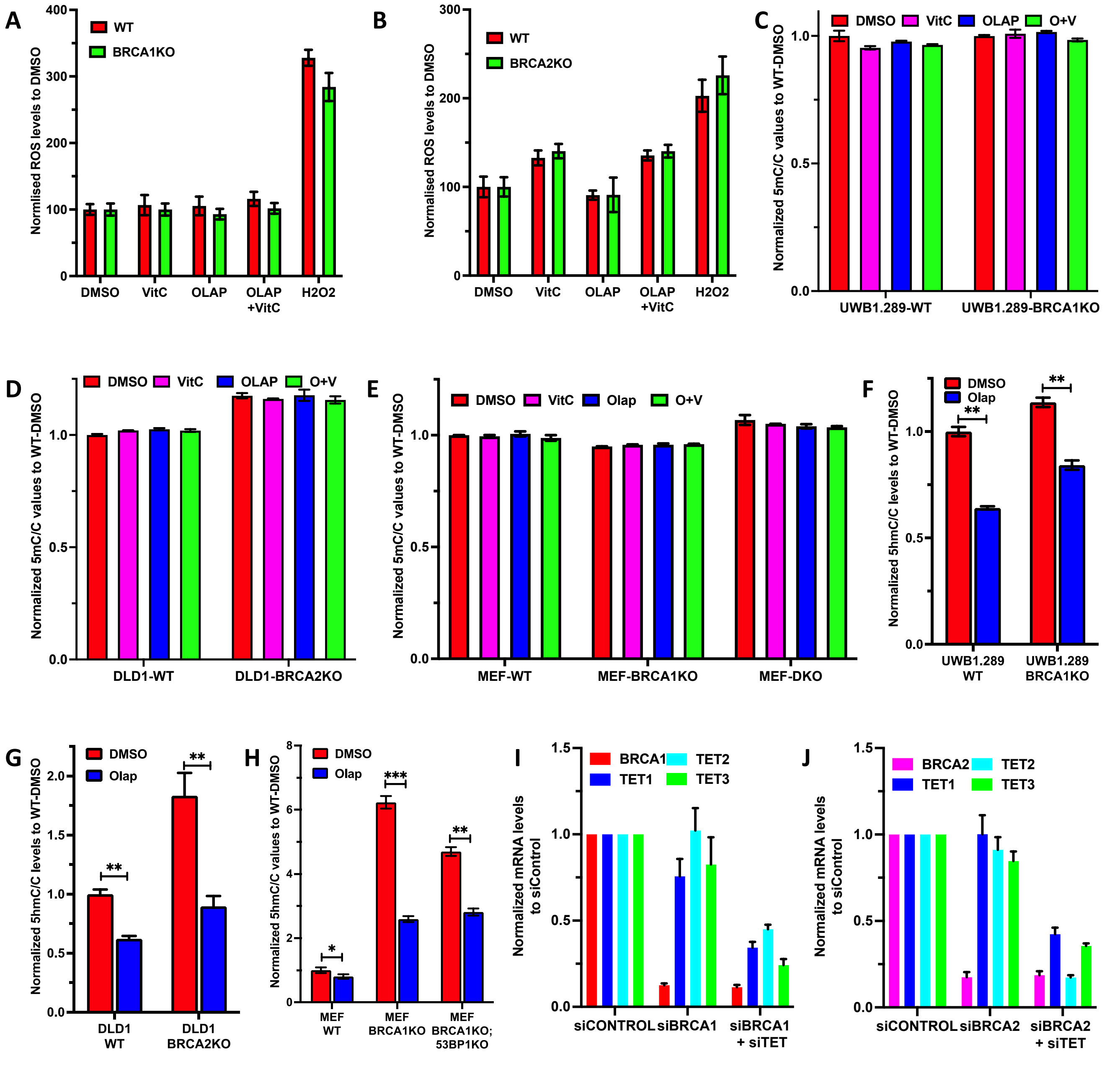

Supplementary Figure 4. 5hmC and VitC traps PARP1 in presence of olaparib on chromatin in BRCA1/2 deficient cells.

A, B. Western blotting of nuclear soluble and chromatin-bound fractions prepared from UWB1.289-WT/BRCA1KO (A), DLD1-WT/BRCA2KO (B) cells upon treatment with VitC (1 mM, 48 hrs) combined with or without olaparib (10 mM, 2 hrs). Blots were probed with indicated antibodies. **C, D.** Western blotting of nuclear soluble and chromatin-bound fractions prepared from UWB1.289-WT/BRCA1KO (C), DLD1-WT/BRCA2KO (D) cells upon treatment with 5hmC (1 mM, 48 hrs) combined with or without olaparib (10 mM, 2 hrs). Blots were probed with indicated antibodies.

Supplementary Figure 4

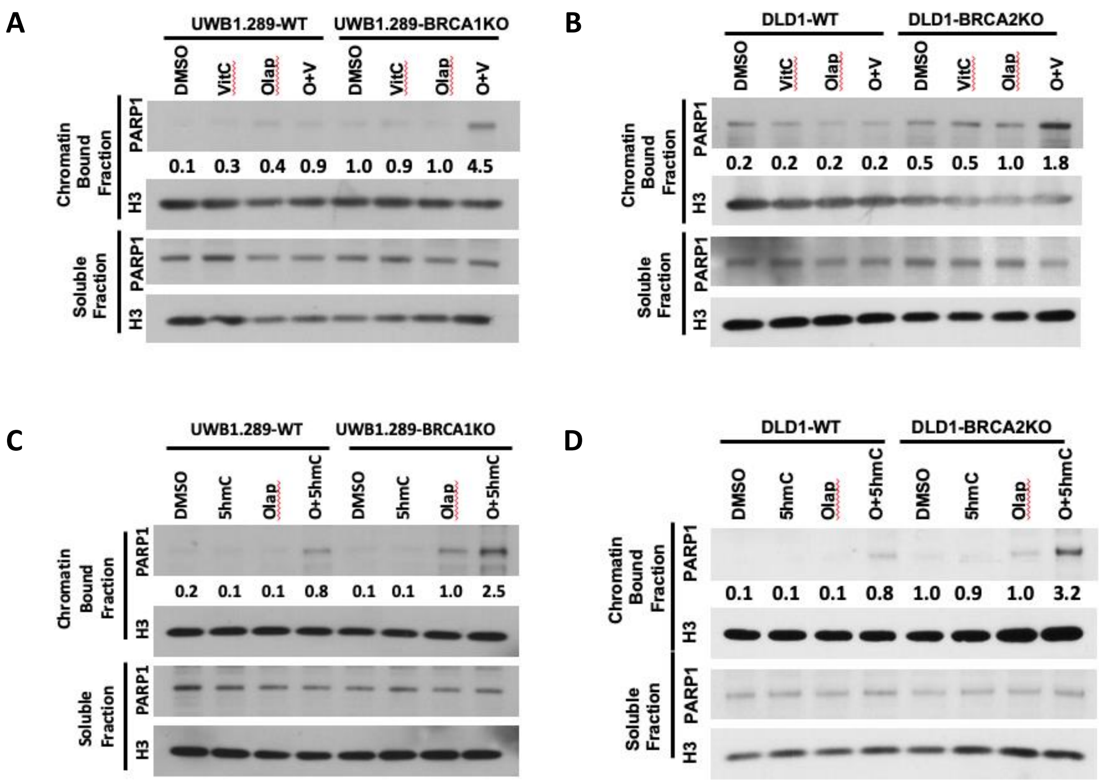

Supplementary Figure 5. 5hmC and VitC increases replication gaps in olaparib treated BRCA1/2 deficient cells.

A, B, C. Representative DNA fibers for IdU tracts with or without S1 nuclease incubation in UWB1.289-WT/BRCA1KO (A) and DLD1-WT/BRCA2KO (B) and WT/*Brca1Δ11*/*Brca1Δ11;53Bp1KO* (DKO) MEF (C) cells upon treatment with 5hmC (1 μM, 48 hrs) and olaparib (100 nM, 48 hrs) or both. **D, E, F.** Representative DNA fibers for IdU tracts with or without S1 nuclease incubation in UWB1.289-WT/BRCA1KO (D) and DLD1-WT/BRCA2KO (E) and WT/*Brca1Δ11*/*Brca1Δ11;53Bp1KO* (DKO) MEF (F) cells upon treatment with VitC (1 mM, 48 hrs) and olaparib (100 nM, 48 hrs) or both.

Supplementary Figure 5

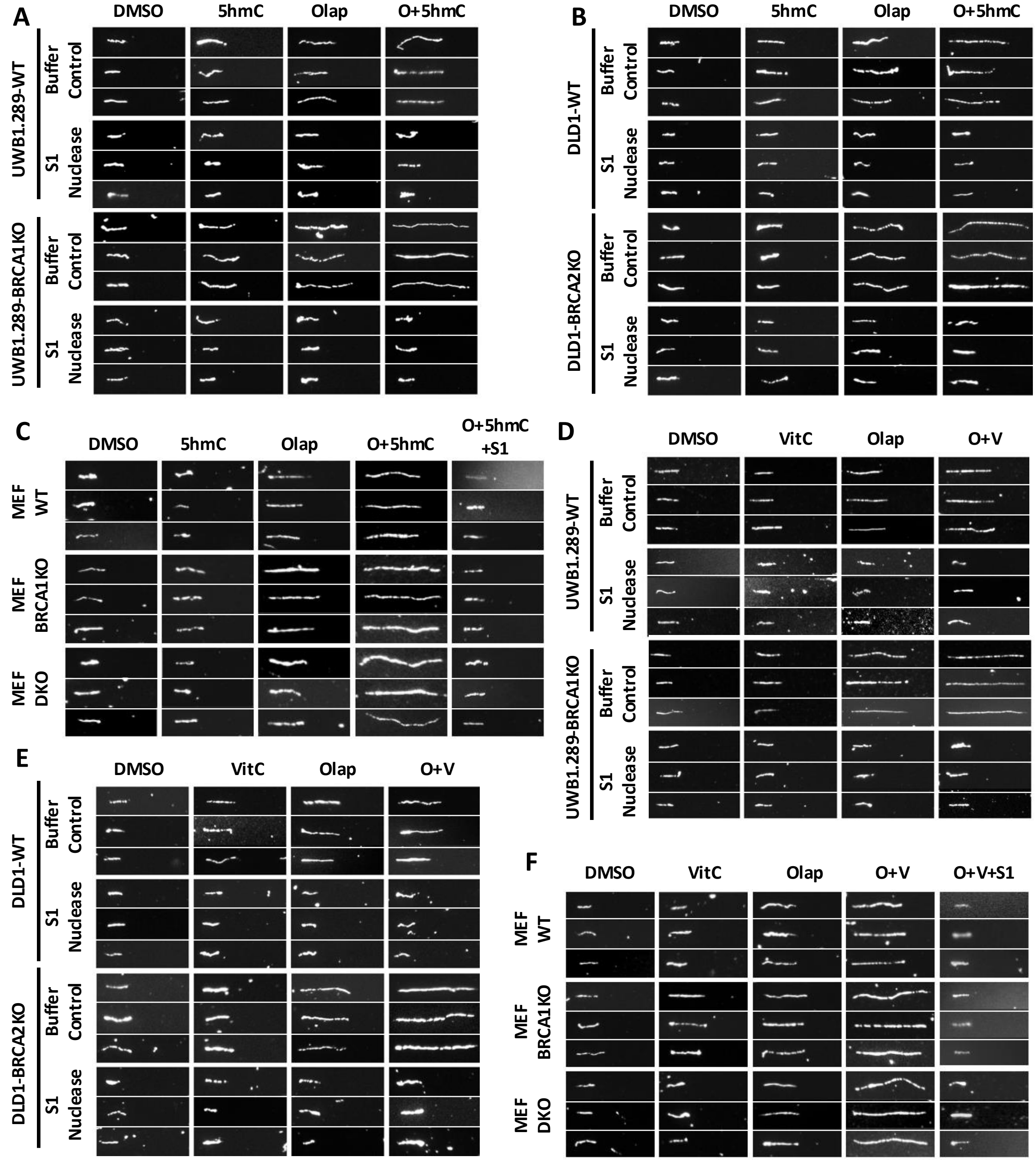

Supplement: Supporting information [file mmc1.pdf]
